# Hydrogel-Based Colloidal Photonic Crystal Devices for Glucose Sensing

**DOI:** 10.3390/polym12030625

**Published:** 2020-03-09

**Authors:** Wenwei Tang, Cheng Chen

**Affiliations:** 1 Modern Service Department, College of International Vocational Education, Shanghai Polytechnic University, Shanghai 201209, China; tangww@sspu.edu.cn; 2 School of Environmental and Materials Engineering, College of Engineering, Shanghai Polytechnic University, Shanghai 201209, China; 3 Research Center of Resource Recycling Science and Engineering, Shanghai Polytechnic University, Shanghai 201209, China

**Keywords:** colloidal crystal, photonic crystal, hydrogel, glucose sensor

## Abstract

Diabetes, a common epidemic disease, is increasingly hazardous to human health. Monitoring body glucose concentrations for the prevention and therapy of diabetes has become very important. Hydrogel-based responsive photonic crystal (PC) materials are noninvasive options for glucose detection. This article reviews glucose-sensing materials/devices composed of hydrogels and colloidal photonic crystals (CPCs), including the construction of 2D/3D CPCs and 2D/3D hydrogel-based CPCs (HCPCs). The development and mechanisms of glucose-responsive hydrogels and the achieved technologies of HCPC glucose sensors were also concluded. This review concludes by showing a perspective for the future design of CPC glucose biosensors with functional hydrogels.

## 1. Introduction

In 1987, Yablonovitch and John proposed the concept of photonic crystals (PCs) based on the properties of semiconductor crystals and electron band gaps [1,2]. As so-called metamaterials in modern photonics, PCs possess specific periodic structures with different dielectric constants that generate photonic band gaps (PBGs), and thus can manipulate the propagation of photons. Artificially constructed PC materials have ordered structures in one, two, or three dimensions (1D, 2D, and 3D) with different refractive indices. PCs can be fabricated by a “top-down” strategy that can usually obtain a regular structure with few defects [3,4,5], whereas “bottom-up” approaches use nanoscale materials to form specific units by aligning and assembling methods. Responsive PCs have tunable PBGs that when induced by external stimuli could be fabricated by incorporating the response materials with PCs. To achieve this, stimuli-responsive hydrogels are used as substrates that are coupled with the PC structure according to specific needs.

Diabetes is a metabolic disease characterized by glucose, protein, fat, and other metabolic disorders that occur due to hypofunction of islets of Langerhans and insufficient insulin secretion [6]. It also causes serious complications, such as heart disease, renal failure, cerebrovascular disease, blindness, and so on, which greatly endanger people′s health. Accompanying the development of society, people′s eating habits have gradually diversified and their labor intensity has relatively reduced, causing an increase in the diabetes population. It is important to monitor blood glucose (BG) concentrations for the prevention as well as the therapy of diabetes. As BG in diabetics can undulate significantly throughout the day, it should be monitored frequently. The commonly used self-analysis of BG levels requires a tiny blood sample (<1 μL) obtained by a “finger-pricking” collection method; the glucose data should be collected several times a day, which is inconvenient and discontinuous. Hydrogel-based responsive PC materials are noninvasive options for glucose detection. Here, we briefly review glucose-sensing materials and devices comprising hydrogels and colloidal photonic crystal (CPC) materials, which have been extensively studied because of their unique optical properties and functionalities. The review is organized as follows. The construction of 2D/3D CPCs and 2D/3D hydrogel-based CPCs (HCPCs) is described in Section 2. Section 3 introduces the development and mechanisms of glucose-responsive hydrogels and the achieved technologies of HCPC glucose sensors. A summary and perspective are given in Section 4.

## 2. Preparation of CPCs and HCPCs

PCs with different dimensionally ordered structures are prepared in different ways. Assembled 1D PCs have been investigated as Bragg reflectors [7], whereas 2D/3D PCs have been extensively researched in areas related to displays, sensing, telecommunications, solar energy, and photocatalysis [8,9,10]. The most effective method to obtain periodic PBG structures is by self-assembly technology; such a method utilizes one or more kind(s) of colloidal particles that form periodic array structures by directional alignment under appropriate conditions. Compared with the “top-down” manufacturing process, the self-assembly method has the advantages of high efficiency, low cost, and scalable production. Moreover, self-assembly happens to all things either statically or dynamically [9,11,12]. Colloidal self-assembly is mainly induced by the synergistic effect of macroscopic or microscopic forces such as liquid surface tension, capillary force, van der Waals force, chemical bond, external field, etc. The resulting CPCs from self-assembly have a periodic structure analogous to the crystalline lattice structures, except that the atoms or ions are replaced by colloidal particles and show interesting optical properties and useful applications [13].

### 2.1. CPCs by Self-Assembly of Colloidal Particles

#### 2.1.1. Opaline 3D CPCs

**Figure 1:**
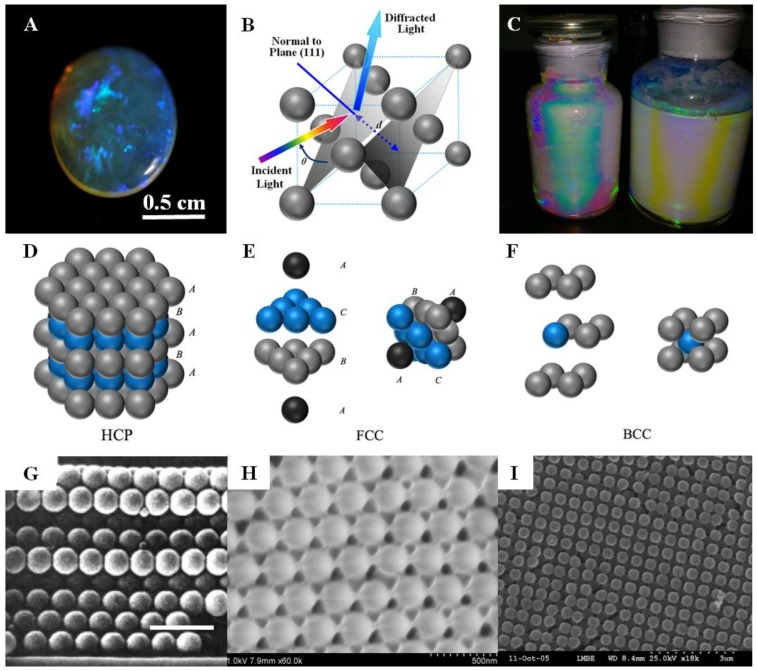
(**A**) Photograph of a crystal opal gemstone from South Australia; (**B**) illustration of the diffraction from the (111) planes of the opal that follows Bragg’s law; (**C**) opaline non-close-packed (NCP) colloidal photonic crystals (CPCs) self-assembled from polystyrene (PS) colloids that were stored in jars; schematic illustration of (**D**) hexagonal-close-packed (HCP), (**E**) face-centered-cubic (FCC), and (**F**) body-centered-cubic (BCC) structures; SEM images of (**G**) HCP [24] (scale bar is 1 μm), (**H**) slightly NCP FCC, and (I) BCC structures [25].

Opals, naturally occurring CPCs, show iridescent colors (Figure 1A), and the periodic microstructure found in opals consists of silica colloids of 150–350 nm in diameter [14]. In 1968, Werner Stöber developed a chemical process for the synthesis of monodisperse silica sphere colloids with controllable and uniform sizes [15]. The Stöber process has opened up a path for fabricating silica-based materials, especially in replicating opals by self-assembly of silica or silica-based hybrid colloids. Other inorganic oxide colloids, such as ZnO and TiO_2_, and polymeric colloids, such as polystyrene (PS)-, poly(methyl methacrylate) (PMMA)-, and PS/PMMA-derived copolymer spheres (e.g., poly(styrene-methyl methacrylate-acrylic acid) (P(St-MMA-AA))), were utilized to fabricate opaline structures [16,17]. The properties of the colloids can be further modified by coatings with various chemical compositions at different thicknesses surrounding the spheres. With uniform coatings, the obtained core–shell colloids can maintain their narrow size distribution and thus self-assembly can readily occur.

The self-assembly process usually relies on the gravity, capillary, centrifugal, electrostatic, or magnetic field that causes the polymer colloidal particles to gather into a close-packed or non-close-packed (NCP) structure. For the close-packed structure, in principle, the CPC should retain a face-centered-cubic (FCC) structure as it is the most thermodynamically stable phase. Self-assembly in a gravity field seems to be the simplest way to obtain CPCs, and although their preparation process is relatively simple, such methods actually involve several complex processes, such as gravity sedimentation, Brownian motion, solvent evaporation, and crystallization (i.e., nucleation and crystal growth) [18]. The parameters of self-assembly, including the size, uniformity, and density of the colloids, and sedimentation rate, must be carefully selected to grow highly ordered CPCs with long-term order. The main disadvantages of this method include insufficient control of the surface morphology and array thickness of the CPCs, long preparation time, and polycrystalline structures. Moreover, the surface of the substrate must be flat, clean, and chemically homogeneous to generate a high-quality array with relatively large domain sizes greater than hundreds of micrometers. Therefore, HCP structures and other defects often occur due to the tiny difference in the Gibbs free energy between the two phases (≤10^−3^ *RT* per particle). Thus, planar stacking dislocations and polycrystalline structures (mixtures of FCC and HCP) may occur when the assembly process is perturbed [19,20,21,22]. On the other hand, for NCP conditions, the formed CPC lattice morphology could be an FCC structure with a high volume fraction of polymer colloids, whereas the body-centered-cubic (BCC) structure was observed at a low volume fraction of polymer colloids [23]. The crystal structures of HPC, FCC, and BCC are illustrated in Figure 1.

The typical opaline CPCs with different colors are shown in Figure 1C. The diffraction wavelengths and the diffraction colors are predicted through Bragg’s law:

*λ*_0_ = 2*n_a_d_hkl_*sin*θ* (1)

where *λ*_0_ is the diffraction wavelength in air, *d_hkl_* is the interplanar crystal spacing, *n_a_* is the average refractive index of the system, and *θ* is the observation angle. For CPCs, *d_hkl_* is in nanoscale, which implies the diffraction wavelengths of CPCs may cover the ultraviolet (UV), visible, and near-infrared (NIR) regions. Until now, opaline CPCs were not expected to exhibit full PBGs; nevertheless, they were easy to prepare, and derived structures as well as functionalized devices were developed.

#### 2.1.2. Inverted Opaline 3D CPCs

Theoretical studies show that the Brillouin zone of the FCC lattice structure is the most spherical to form PBG. However, the resulting band gap of opaline CPCs is only a pseudo-gap. Fortunately, they can still be used as templates to create new structures, and complete band gaps (stop-bands) might be achieved. The inverted opaline (inverse opal (IO)) structures can be directly replicated from opal-derived materials by utilizing inorganic materials, such as SiO_2_, SiC, ZrO_2_, and TiO_2_ [26,27,28], or polymers such as polyurethane (PU) [29], which have attracted considerable interest in recent years owing to their novel optical and electronic properties; high specific surface areas; and potential applications in the realms of PBG materials, power sources, and catalysts.

**Figure 2:**
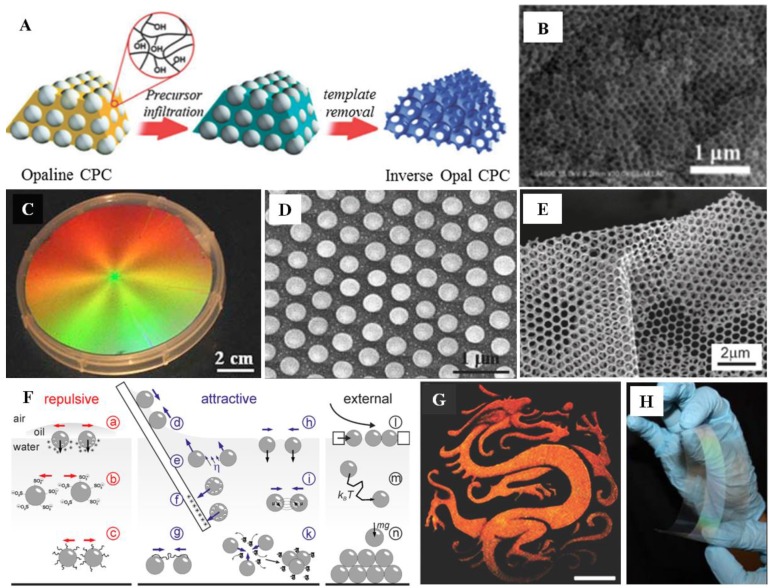
(**A**) Schematic diagram of the fabrication of inverse opals; (**B**) SEM of TiO_2_ inverse opal (IO) [30]; (**C**) an optical photograph of a 2D CPC prepared by spin coating; (**D**) high-resolution SEM photographs of an NCP 2D CPC [31]; (**E**) SEM images of an Ag_2_S nanonet derived from 2D CPCs as a template [32]; (**F**) schematic representation of various forces and interaction motifs for colloidal particles during self-assembly [33]; (**G**) angle-independent dragon patterns on polyethylene glycol terephthalate (PET) film by spray coating (scale bar is 5 cm) [34]; (**H**) 2D CPC prepared by the roll-to-roll method [35].

As shown in Figure 2A,B, the inversion of opaline structures involves the infiltration and removal of the opaline templates. The interstices between the colloids are first filled with different solutions or precursors that have the crucial role of matrices, and then the colloids are removed by dissolution or calcination, while leaving air voids in the matrices, and the resulting systems are also CPCs. However, there are difficulties associated with forming ordered inverse opals using this method. For instance, the filling volume is limited owing to the close-packed structure of the CPC templates, and several infiltration cycles are required to produce a homogeneous, robust inverted opaline structure due to the hydrophobic character of polymer-based CPC templates [30].

#### 2.1.3. D CPCs

Different to 3D CPCs, colloids that are self-assembled in the planar direction result in a monolayer structure, and are thus called 2D CPCs. The commonly used self-assembly methods for preparing 2D CPCs are spin coating and interface spreading. Spin coating is a process where colloidal particle suspensions are spread on a substrate at a high shear rate, and colloids are rapidly and densely assembled on the surface of the substrate material under the action of centrifugal force with the rapid volatilization of suspension solvents. It was verified that the regularity and stacking layers of CPCs obtained by spin coating are mainly affected by factors such as rotation speed, suspension concentration, affinity between substrate and solvent, etc. [36].

Interface spreading first injects the solvent dispersed colloids into the surface of the substrate material; a monolayer film with regular arrangement can be formed at the gas–liquid or liquid–liquid interface due to the surface tension difference. The self-assembly behavior of spherical colloid suspensions was first studied in 1954 [37]. On this basis, assemblies of single-layer CPCs were obtained by spreading colloids on a horizontal substrate [38]. During the spreading process of colloids, continuous solvent evaporation caused the originally randomly dispersed colloids to spontaneously aggregate under the co-effects of capillary force and convection transmission to form a close-packed arranged monolayer structure. Next, a new method to continuously form hexagonal close-packed high-quality 2D CPCs on a vertical substrate was developed [39]. This method utilized Marangoni effects: with the gradient surface tension, the solvent spread rapidly on the liquid surface, driving colloids to disperse and spread rapidly outward at the interface at the same time, thus forming densely packed and long-range-ordered monolayer crystals [40]. Besides, other feasible self-assembly methods, such as injection or roll-to-roll printing, can be used to prepare 2D CPCs in a large area with bright iridescent structural colors [35,41,42]. Moreover, a 2D CPC with IO structure could also be obtained by etching of colloids: an Ag_2_S self-supporting nanonet film (Figure 2E) was prepared on a large scale by gas–liquid interface etching combined with a gas diffusion reaction. The mesh of the film has a regular and orderly periodic structure, so it has the characteristics of a 2D CPC [32].

### 2.2. Hydrogel-Based CPCs (HCPCs)

Hydrogels are hydrophilic, 3D, semi-open and water-insoluble network structures formed with physical or chemical methods by polymers that contain significant amounts of a water mobile phase. The hydrophilicity of hydrogels arises from the hydrophilic pendant groups of the polymeric molecules such as alcohols, carboxylic acids, and amides, and some hydrogels can undergo a significant volume phase transition (VPT) in response to environmental stimuli. For instance, pH-sensitive polymers usually contain acidic or basic groups that can gain or lose protons in relation to the pH change. Thermosensitive hydrogels swell/shrink according to temperature change due to the presence of pendant groups, such as methyl, ethyl, and propyl, that are responsive to temperature. Other sensitivities such as photo-, electro-, gas-, and metal-response abilities ensure the wide areas of application of hydrogels [43].

The combination of CPCs and the hydrogel system can transform the VPT responses to temperature, pH, light, electric field, pressure, biomolecules, and other factors into a shift of PBG in the CPCs, which can be detected relatively easily by optical methods. Visual detection can be realized as the PBG (and/or its change) of the hydrogel/CPC system is modulated in the visible light region.

**Figure 3:**
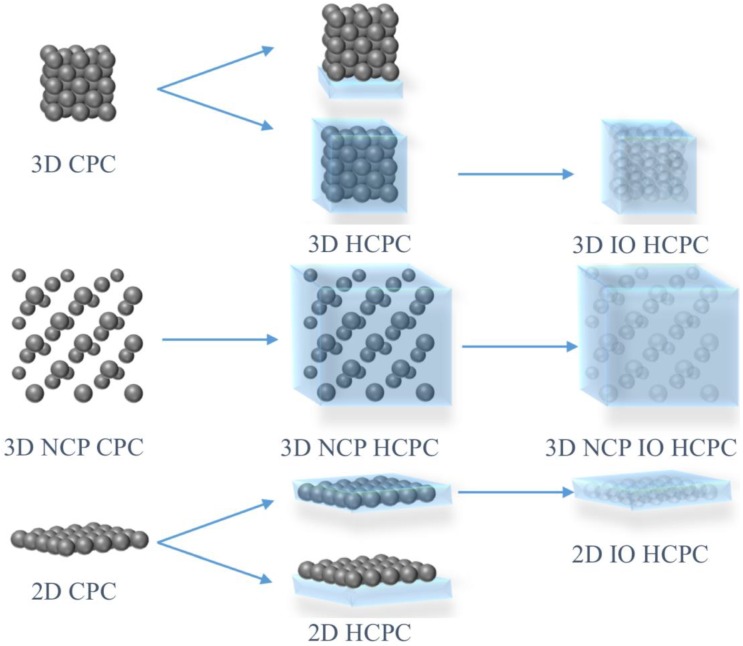
A brief illustration of the construction of hydrogel-based colloidal photonic crystals (HCPCs) with different combination styles. The upper style, in which the colloidal photonic crystal (CPC) is assembled onto the hydrogel, is rarely reported [45,46].

According to Bragg’s law, the commonly utilized factors to change the PBG of HCPCs are as follows [44]: (1) The average refractive index, *n_a_*, which is determined by the refractive index of each substance in the system and its volume fraction. The hydrogel network or CPC itself can adsorb specific substances, and thus can cause a change in *n_a_*, and then shift the PBG; (2) The change in lattice constant (simplified here, the interparticle distance of the colloids), which usually follows the change in hydrogel volume as a response of gel to external stimuli; (3) The ordering of the colloids, including anisotropic and isotropic effects. For anisotropic changes, such as mechanical stretching, compressive pressure, and magnetic force, the symmetry of CPCs could be disturbed and thus change the diffraction properties. For isotropic changes, such as the scattering ability of the CPC being affected by the size change of the colloids, the diffraction intensity could be tuned. Combination methods of HCPCs with different styles are illustrated in Figure 3.

Based on these methods, a series of HCPC-sensing materials were developed in the past 25 years since Asher’s pioneering work [47]. This first HCPC optical material comprises a 3D NPC CPC of 150 nm PS colloids embedded in hydrogel via UV polymerization, and the hydrogel comprises a co-monomer of *N*-vinylpyrrolidone and acrylamide, and *N,N*’methylenebis(acry1amide) as a cross-linker. The diffraction color of the HCPC could be shifted by applying a uniaxial strain to the HCPC film. Moreover, by removing the CPC from the HCPC, the IO HCPC could be obtained, which provides a larger VPT as the colloid voids are replaced by water [48]. As the NPC CPC is formed by self-assembly, which relies on the electrostatic repulsion of the colloids to form a 3D periodic structure, it is very sensitive to ions. A tiny amount of ionic impurity will disturb the meta-stable CPC array, and thus the monomers for HCPC preparation are limited to nonionic. To conquer this, physically cross-linked poly(vinyl alcohol) (PVA) hydrogel was utilized to form HCPCs [49,50], and then ionic hydrogels could be added to the system by secondary UV polymerization [51]. Optionally, the PVA could be dissolved through thermal treatment and the ionic HCPC maintained [52].

So far, HCPCs have become a hot spot in the research field of photonic materials due to their great application potential in biological separation, bionic materials, environmental detection, medical diagnosis, displays, anti-counterfeiting, various chemical or physical sensors, and other fields.

## 3. HCPC Glucose-Sensing Materials and Devices

### 3.1. History of HCPC Glucose Sensor Materials

As previously mentioned, the optical response of HCPCs is mainly due to the hydrogel volume response to certain stimuli. For the glucose-sensing purpose, glucose-sensitive hydrogels are used as the matrices of HCPCs. Glucose-sensitive hydrogels are hydrogels that can recognize glucose molecules in the environment and swell/shrink according to the glucose concentration. Glucose-sensitive HCPCs can be mainly divided into three types due to their sensitive mechanisms: glucose oxidase (GOx)-immobilized HCPC, concanavalin A (Con A)-containing HCPC, and boronic acid (BA)-derived HCPC.

#### 3.1.1. GOx-Immobilized HCPC

The first HCPC for glucose sensing was reported by attaching the enzyme GOx to a polyacrylamide (PAM) HCPC [53]. GOx can catalyze and oxidize glucose into gluconic acid and generate H_2_O_2_ at the same time, thus reducing the pH value of the system, resulting in an increase or decrease in the swelling degree of the hydrogel:

GOx(ox) + glucose → GOx^−^(red) + gluconic acid + H^+^, (2)

H^+^ + GOx^−^(red) + O_2_ →H_2_O_2_ +GOx(ox), (3)

**Figure 4:**
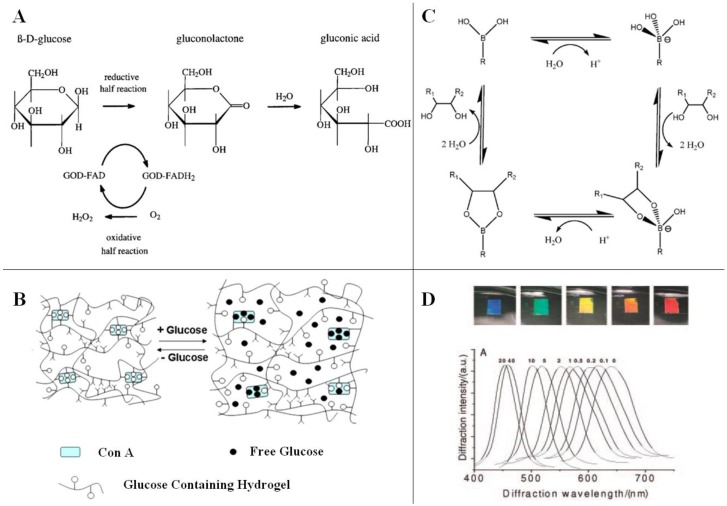
An illustration of the mechanisms of glucose response: (**A**) reaction between GOx and glucose [54]; (**B**) reversible glucose binding by Con A [56]; (**C**) boronate–glucose complex [57]. (**D**) Glucose concentration change resulted in an osmotic pressure that caused the hydrogel to swell, which red-shifted the Bragg diffraction wavelength and caused a change in diffraction color [58].

In actuality, this reaction can be divided into a reductive and an oxidative step, as shown in Figure 4. In the reductive half reaction, GOx catalyzes the oxidation of β-D-glucose to D-glucono-δ-lactone, which is non-enzymatically hydrolyzed to gluconic acid. Subsequently, the flavine adenine dinucucleotide (FAD) ring of GOx is reduced to FADH_2_. In the oxidative half reaction, the reduced GOx is reoxidized by oxygen to produce H_2_O_2_, which can be decomposed into water and oxygen by catalase [54]. Although the sensitivity was improved by further research [55], the utility of the GOx HCPC for measuring glucose was limited because of (1) the short usage due to the storage difficulty and irreversible reaction of GOx; (2) its failure at high ionic strength; and (3) its response was also dependent on the concentration of oxygen, accompanying the production of H_2_O_2_, which could be problematic for many applications.

#### 3.1.2. Con A Complex HCPC

Another HCPC utilizes a specific phase-reversible interaction between glucose and Con A. Plant lectins, such as Con A, can specifically bind monosaccharides with four glucose binding sites and function as cross-linkers for the glucose-containing polymer chains within the hydrogel. Such hydrogels will swell due to competitive desorption of polymer-bound glucose from the binding sites of Con A by external free glucose, and thus red-shift the diffraction wavelength [59].

Dai proposed a glucose sensor based on this idea [60]. A glucose-immobilized PAM HCPC was coupled to Con A, and the free glucose concentration was analyzed through the PBG shift due to the VPT of hydrogel. Superior to the GOx enzymatic HCPC, the glucose detection of the Con A HCPC will not be affected by the ionic strength as the VPT only relies on the cross-linking density of the polymer chains. Despite the fact that only 20 nm red-shifting was found when 25 mM glucose was added in buffer solution, the Con A combined HCPC can also be used in insulin controlled release systems, providing an option for glucose concentration monitoring.

#### 3.1.3. BA-derived HCPC

GOx and Con A are natural proteins that are both very sensitive to environmental changes and will cause an immune response when exposed in the body. BA and its different derivatives can covalently and reversibly bond with the diol moieties of glucose, and various boronate complexes (trigonal/tetragonal) could form depending on the pH value of the medium. Compared with natural proteins, BA derivatives have good stability and do not cause immune responses of organisms due to their fully synthetic system. Theoretically, complexation between BA and glucose generates Donnan osmotic pressure, resulting in a VPT of the hydrogel that will swell/shrink to different extents according to the glucose concentration.

Similar to Con A, a borax competitive binding glucose-sensing HCPC that could respond to blood glucose was proposed [61]. Glucose competes with the binding of PVA diols to borate ions. The diffraction wavelength blue-shifted ~62 nm when the glucose concentration increased from 0 to 40 mM. Although this sensor showed a good sensing range for glucose in PBS, there was little response to glucose at physiological pH due to the low affinity of boric acid and glucose; the effective p*K*_a_ for boric acid within the hydrogel was ~8.4, thus being unsuitable for in vivo monitoring applications.

The most investigated functional sensing unit for glucose is phenylboronic acid (PBA), which can form reversible covalent complexes with molecules containing OH groups. As a Lewis acid, PBA exists in aqueous solution in two forms: charged tetrahedral boronate and uncharged trigonal BA. The ionized PBA can stably bind with glucose molecules, whereas the uncharged trigonal PBA-glucose complex is unstable at a given pH value [62]. An increasing concentration of glucose drives the formation of dynamic covalent bonds and generates PBA-glucose complexation, which effectively reduces the p*K*_a_ of the trigonal BA. This dynamic equilibrium allows more glucose to bind to the PBA-functionalized hydrogel, and as a result, the hydrogel swells.

Following this, the 3-aminophenyl-boronic acid (APBA) pendent group was grafted into the backbone of PAM with the help of 1-[3-(dimethylamino) propyl]-3-ethylcarbodiimide hydrochloride (EDC) [63]. This PBA-modified PAM HCPC showed a red-shift of the diffraction peak with the increasing concentration of glucose in Tris buffer at pH 8.5. However, the sensor became unresponsive to glucose at pH < 7 and pH > 9.5 as all of the boronic acids titrate to boronates and the sensor swells. Thus, this sensor only worked at low ionic strength. Nevertheless, it was the first time that the HCPC was proved to generate a distinguishable color change according to glucose concentration.

To solve the ionic problem, a poly(ethylene glycol) (PEG)-functionalized HCPC was then developed with improved ionic stability, which could be operated at physiological pH and ion strength [64]. Glucose binding caused the formation of a supramolecular bis-bidentate glucose–boronic acid complex, which was stabilized by PEG and sodium cations. A two-way response of glucose was observed: (1) At low glucose concentrations, the glucose cross-linked two boronates and the PEG moieties localized the cations close to the BA. Thus, the complex between glucose and the two boronates was electrostatically stabilized, resulting in the shrinkage of the HCPC, and the diffraction blue-shifted. (2) At high glucose concentrations, the cross-linking of glucose and boronates dissolved due to the saturation of the boronate sites. Consequently, the HCPC swelled and the diffraction red-shifted. This work further clarified the mechanism of VPT in the glucose response process of PBA-HCPCs, and it also demonstrated the potential for in vivo glucose monitoring by such photonic materials. However, the data could be easily confused as the diffraction wavelength shift was not monotone. Meanwhile, an APBA-functionalized poly(2-hydroxyethyl methacrylate) (PHEMA) IO HCPC was developed to enhance the sensing ability. The IO structure ensured the response to glucose at both low and high ion concentration as well as a faster response time [65]. It was also reported that poly(N-isopropylacrylamide) (PNIPAM) and PBA were copolymerized to make an IO HCPC that was dual-sensitive to thermal and glucose stimuli, which provides a wide range of ideas for future researchers [66].

Subsequently, 4-amino-3-fluorophenylboronic acid (AFPBA) with low p*K*_a_ was utilized as the molecular recognition element for further improving the sensitivity and selectivity of the glucose response, and the HCPC could respond to glucose concentrations of 0–40 mM with detection limits of approximately 1 μM in artificial tear fluid (ATF) [58]. A comparison of glucose-sensing properties could be found in Table 1. The HCPC sensor had good anti-interference performance against other sugars and the major components in ATF. The demonstrated low sensing range is suitable not only for sensing subcutaneous glucose levels, but also for measuring tear glucose levels (0.16 ± 0.03 mM mean in normal individuals, and 0.35 ± 0.04 mM mean in diabetics) [67,68]. Thus, a wearable contact lens glucose sensor with colorimetric measurement was successfully proposed. Recent research works incorporated molecular imprinting technology to improve the specific recognition of glucose molecules, and thus improve the sensitivity and accuracy of the sensor [69,70,71,72].

### 3.2. Current and Emerging Devices

#### 3.2.1. D HCPC Glucose Sensors

**Figure 5:**
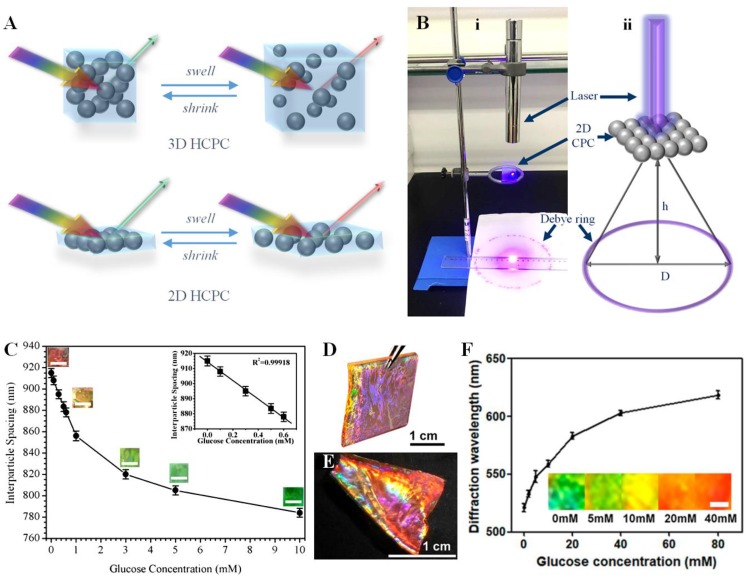
(**A**) Comparison between 3D and 2D HCPCs: 3D HCPCs are formed by cross-linking hydrogel networks around a 3D colloid array, whereas 2D HCPC-sensing devices are formed by attaching a 2D CPC onto a hydrogel containing functionalized recognition groups. (**B**) Debye diffraction ring characterization: (i) the photograph shows the Debye diffraction ring resulting from a 2D CPC under 445 nm wavelength laser light, and (ii) a schematic diagram of Debye diffraction ring detection [74]. (**C**) Interparticle spacing change of a 2D HCPC with different glucose concentrations; the photographs show that the forward-diffraction color changed from red, through yellow, to green, and the inset shows a linear stop-band shifting with the increasing glucose concentration (0.1−0.6 mM) [80]. Photographs of (**D**) 2D Au CPC and (**E**) 2D Au HCPC that showed high diffraction efficiency. (**F**) Glucose concentration dependence of the diffraction wavelength of the 2D Au APBA -PAM-PVA HCPC device; inset shows photographs of the 2D HCPC film at different glucose concentrations [81].

The 2D HCPC is one of the simplest but most effective colorimetric sensing devices. Its thinnest monolayered crystal structure provides high photon refraction efficiency and provides more space for the extension of coupled hydrogels. The main differences between 2D HCPCs and 3D HCPCs are illustrated in Figure 5A. As the 2D CPC has ordered repeating units around hundreds of nanometers, this periodic structure is able to strongly diffract visible light; theoretically, only ~20% energy of the incident light was lost as it went through the particles and then diffracted. Iridescent diffraction colors could be observed from 2D CPCs, which originate from the periodic structure of ordered repeating units around hundreds of nanometers of the assembled colloids. A diffraction of six-spot symmetry will be shown when illuminated with monochromatic laser light if the colloids form a perfect hexagonal 2D CPC. Actually, the 2D CPC exposed to a laser beam is randomly oriented as the crystallites are significantly smaller than the diameter of the beam, thus many symmetry spots with the same diffraction diameter will form a ring, and such a pattern is called a Debye diffraction ring [73]. As shown in Figure 5B, a Debye diffraction ring was observed as the 2D CPC sample was excited along its normal with a 445 nm laser pointer [74]. The spacing of the colloids in the 2D CPC can be calculated by the following equation, and the related parameters can be clarified as:

(4) $$d=\frac{4\lambda_{laser}\sqrt{{\left(D/2\right)}^{2}+h^{2}}}{\sqrt{3}D}$$

where *λ_laser_* is the incident wavelength, *d* is the nearest neighboring colloids’ spacing, h is the distance between the 2D CPC sample and the screen, and D is the diameter of the Debye diffraction ring on the screen.

The advantages of 2D HCPC-sensing devices mainly include (1) independent fabrication of 2D CPCs and hydrogels, as the monolayered colloid arrays could be either attached onto the hydrogel surfaces or embedded into the hydrogel during polymerization; (2) the response of the sensing device could be determined by measuring the Debye ring instead of using a fiber spectrometer; and (3) the readout of the 2D HCPC-sensing device is reliable since the diffracted light from the 2D structure is not affected by the change in refractive index during measurement [75].

The potential for glucose sensing by 2D HCPC devices has been investigated since the technology for the preparation of 2D CPCs on a large scale was developed. For a glucose-sensing motif, a highly selective glucose-sensing protein 2D HCPC was reported that can shrink due to protein conformational change induced by ligand binding [76]. Although the mechanism of this technology is different from that of the above-mentioned methods, the preparation time and response time for such a device was too long for point-of-care detection. In view of the diversity of glucose sources in the human body, in addition to BG sensing, there are also different studies on the detection of glucose in urine, tear fluid, saliva, etc., which also made significant progress. A 2D HCPC with APBA-modified polyacrylamide-*co*-acrylic acid (PAM-AA) hydrogel for urine glucose sensing was reported, which can avoid the urine-interfering elements, but the diffraction color change could not be distinguished at low glucose concentration [77]. Following this, research with improved color response was reported for clinical urine sample tests [78]. A 2D HCPC tear glucose sensor was prepared, which exhibited rapid response to glucose and reached binding equilibrium within 3 min, and the structure color of the sensor shifted from red to green as the glucose changed from 0 to 20 mM. However, the response in the physiological range could not be distinguished by the naked eye, which limited its application for in vitro sensing [79]. Another 2D HCPC glucose sensor utilized 4-boronobenzaldehyde (4-BBA)-functionalized PVA hydrogel as the sensing matrix, ensuring the high ionic sensing of glucose under physiological concentrations [80]. Such a device indicates hyperglycemia as its diffraction color changes from red to yellow, and even to green in ATF, and the sensing process is fully reversible, as shown in Figure 5C. Similarly, an APBA-PAM-PVA system was utilized with an Au colloid array that improves the diffraction efficiency, as shown in Figure 5D–F; interestingly, this 2D HCPC device showed good linearity and only red-shift was observed as the glucose concentration increased [81]. The comparison of glucose-sensing properties of 2D HCPCs could be found in Table 2.

So far, different optimization methods have been used to improve the sensitivity of 2D HCPCs. Compared with the dry chemistry method for qualitative analysis, such 2D HCPC sensor devices are more accurate and cost-effective, and provide a new approach to detect glucose in various body fluids with higher sensitivity and less interference.

#### 3.2.2. Contact Lens

**Figure 6:**
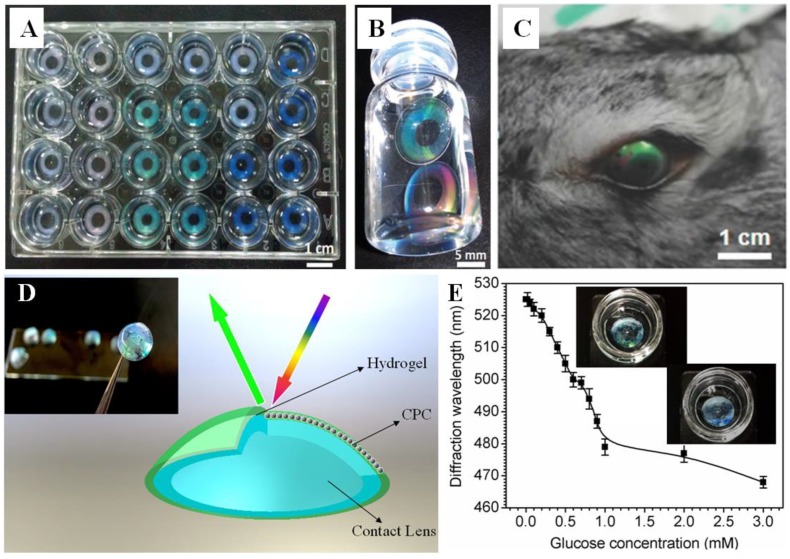
(**A**,**B**) Photographs of structural colored PHEMA contact lenses [88]. (**C**) Photograph of a rabbit wearing a structurally colored contact lens sensor [92]. (**D**) Diagram and photograph (insert) of a HCPC contact lens [6]. (**E**) The response of a PVA HCPC contact lens at low glucose concentration; insert shows a photograph of the color changed lens sample [93].

The contact lens is one of the competitive candidates for noninvasive continuous physiological glucose monitoring [82,83,84,85,86,87]. A concept using contact lenses to detect glucose in tear fluid was proposed shortly after the study of HCPC sensors [66]. Limited by the toxicity, wearability, and reliability of hydrogels, as well as the difficulty of embedding a color-diffractional CPC within the lens, there has been little progress until recently. A HCPC contact lens was reported showing a bright structural color cosmetic effect without dyes or pigments [88]. The PHEMA hydrogel utilized in the HCPC is the common material of contact lenses, which showed excellent biocompatibility with improved proliferation viability, as shown in Figure 6A,B. The pupil area of the lens was transparent due to a coffee-ring effect that would not affect the vision on the light transmission during wearing. Such a device might inspire a glucose-sensing contact lens by functionalization of the HCPC lens. A recent work reported a CPC contact lens with the properties of anti-ultraviolet and blue light reflection [89]. More recently, HCPC contact lenses with various functionalities, such as pH monitoring, drug releasing, and eye pressure sensing, have been developed for point-of-care ophthalmic health monitoring [90,91,92].

For glucose detection purposes, a contact lens-based 3D HCPC glucose sensor was developed using PVA physical hydrogel. The initially reported HCPC contact lens was physically cross-linked by the PVA/PEG system covered on a rigid PMMA polymer contact lens. In this design, the PVA/PEG could offer an anti-fouling layer for the penetration of glucose, thus improving the sensitivity to glucose, and the glucose-sensing property was approached by modifying the hydrogel with 4-BBA [6]. A prototype of such a lens is shown in Figure 6D. Figure 6E states the diffraction wavelength shift at a relatively low glucose concentration [93].

In the range from 0 to 1 mM that covers tear glucose concentration, there is an approximate linear correlation between glucose concentration and diffraction wavelength. However, the sensing ability was limited because the volume change of the hydrogel was restricted by the rigid lens; the parameters of the contact lens are customized according to the corneal curvature of 4-month-old New Zealand rabbits, with a curvature of 7.3, and fit with the corneal surface. Despite the exciting design, it remains difficult to measure glucose concentrations in tears using contact lenses. Because of the very low amount of glucose present in healthy control subjects (3.59 mM) and in diabetic subjects (4.69 mM), the tear glucose levels measured appear to vary with the volume of the aqueous tear fraction collected.

This kind of contact lens with structural color has the advantage of high color saturation without dizziness, which implies its possible use in healthcare sensing and functional visual regulation. It is expected that with a certain structural color between red and green, the contact lens can play a role in color separation and discrimination of color difference for color-blind patients.

#### 3.2.3. Other Emerging Devices

Advances aimed at multifunctional materials for photonic healthcare devices have been made for portable healthcare and personalized medicines. For 2D CPC devices, modifications in optical fibers, heterostructures, and molecular imprinting sensors were reported with enhanced properties, which might have enhanced glucose-sensing properties as emerging devices [94,95,96,97]. For 3D CPC systems, the test paper-like CPC materials provide rapid chemical separation and visual response as potential devices in glucose detection [98,99]. As above-mentioned, as recent research for glucose monitoring not only focuses on blood, but also on other body fluids such as sweat, urine, tear, and saliva [100], more and more diversified photonic implantable and wearable devices might appear [101,102].

## 4. Summary and Perspective

Over the past 30 years, photonic materials have attracted huge research interest. Colloidal photonic crystal (CPC) materials diffract visible color with interesting potential applications in dynamically changeable optical properties due to environmental stimuli. Photonic structures can be manufactured with numerous materials including metals, oxides, and polymers via top-down or bottom-up methods. Limited by the current preparation technologies, real photonic technologies still need to be transferred from laboratory to industry. Considering their certain value in environmental science, controlled drug release, chemical detection, and other fields, hydrogel-based CPC (HCPC) sensors are more convenient for semiquantitative detection in laboratory research.

Changes in the CPC array structure can be directly observed by optical methods, and even by the naked eye. CPCs have been extensively investigated as biosensors for analytes such as cells, bacteria, viruses, glucose, and toxins. Glucose-sensing properties and derived sensing HCPC devices for glucose have been researched, and the binding/reaction mechanisms have also been investigated. Recently, CPC devices have been integrated with emerging technologies, such as smartphones and wearable sensors, to improve the convenience and accuracy of detection [85,87,102]. However, the CPC detection of glucose in body fluids still faces multiple challenges, including the cost for precise preparation of CPCs, purification of clinical bio-samples, limited technical capabilities, etc.

Molecularly imprinted technology is an optimal candidate to enhance the sensitivity and selectivity of HCPC sensors. Glucose-imprinted HCPC sensors are expected to be used for the semiquantitative detection of glucose concentration in human tears or urine, so as to provide a noninvasive method for the family prevention and treatment of diabetes. Real-time detection of glucose levels in saliva could also be a possible breakthrough point for HCPC research. Although there is no CPC device for color detection of saliva glucose at present, saliva glucose has the advantage of high correlation with blood glucose, while other body fluids have a lag-of-time relative to blood glucose.

Contact lenses with structural color also provide an option for the development of dynamic sensing devices. Existing attempts include the combination of sensitive HCPCs with rigid lenses for glucose sensing and the in situ preparation of structural color HCPC lenses for drug release or intraocular pressure detection. Dynamic glucose sensing with contact lenses could be realized by utilizing molecularly imprinted technology, and excellent biocompatibility of the selected hydrogel materials must be a prerequisite.

Photonic structures exist in many natural beings, and bionics has become a hot spot for CPC research [103]. Recent studies on biomimicking chameleon skin showed that CPC structures might have more potential applications in military, biomedicine, electroskin, and smart wearable devices [104,105]. Moreover, with the development of material science and technology, especially 3D/4D printing technology [106], there may be more potential for the development of new structures of CPCs. Combined with self-healable or biocompatible hydrogels [107,108], multi-sensitive and renewable HCPCs can be obtained via self-healing processes, and the future design of CPC glucose biosensors still has an exciting future.

## Figures and Tables

**Table 1:** Glucose-sensing properties of early reported HCPCs with different response mechanisms.

Type	Hydrogel	Detection Limit (mM)	PBG Shift (nm)	Linearity Range (mM)	Sensitivity (mM/nm) ^1^	Response Time	Detection Medium	Ref
GOx	PAM	0.1–0.5	~115	NA	NA	<2 min	NA	[57]
	epoxy-PAM	0.1–1	86	0–0.2	430	<5 min	NA	[53]
Con A	PAM/Con A	5–25	20	0–10	~2	NA	PBS	[59]
BA	PVA/borax	0.33–40	~62	0–40	~1.55	<2~3 min	PBS	[60]
	APBA-PAM	0.05–100	128	0–5	~6.2	NA	Tris-HCl	[62]
	APBA-PEG-*co*-PAM	0.2–50	64 B-shift 20 R-shift	0–8 8–50	8 0.48	NA	Tris-HCl	[63]
	APBA-PHEMA	0.1–100	120	NA	NA	~45 S	CHES	[64]
	APBA-PNIPAM	5–20	~83	5–20	~5.53	NA	CHES	[65]
	AFPBA-PEG-*co*-PAM	0.001–40	234	NA	NA	NA	ATF	[66]

^1^ Diffraction wavelength shift upon glucose concentration in the linearity range of HCPCs, calculated according to reference data.

**Table 2:** Glucose-sensing properties of recently reported 2D HCPC devices.

Hydrogel	Detection Limit (mM)	PBG Shift (nm)	Linearity Range (mM)	Response Time	Detection Medium	Ref
protein hydrogel	1.24 × 10^−^^5^-10	20	NA	~20 min	PBS	[76]
APBA-PAM-*co*-AA	0–15	NA	0-1	<230 S	artificial urea	[77]
APBA-PAM-*co*-AA	0.4–53.3	80 (0.1–10 mM)	0.1–2	20 min	Clinical urine	[78]
APBA-PAM-*co*-AA	0–20	NA	NA	<3 min	CHES and ATF	[79]
BBA-PVA	0.1–10	25 (0.1–0.6 mM)	01–0.6	<200 S	ATF	[80]
APBA-PAM-PVA	2–80	120	0–20	NA	CHES	[81]
